# The IL‐21‐TET2‐AIM2‐c‐MAF pathway drives the T follicular helper cell response in lupus‐like disease

**DOI:** 10.1002/ctm2.781

**Published:** 2022-03-28

**Authors:** Haijing Wu, Yaxiong Deng, Di Long, Ming Yang, Qianwen Li, Yu Feng, Yongjian Chen, Hong Qiu, Xin Huang, Zhenghao He, Longyuan Hu, Heng Yin, Guangdi Li, Yunkai Guo, Wenhan Du, Ming Zhao, Liwei Lu, Qianjin Lu

**Affiliations:** ^1^ Department of Dermatology Second Xiangya Hospital Hunan Key Laboratory of Medical Epigenomics Central South University Changsha China; ^2^ Department of Public Health Central South University Changsha China; ^3^ Department of Otolaryngology Head and Neck Surgery Second Xiangya Hospital Central South University Changsha China; ^4^ Department of Pathology and Center for Infection and Immunology The University of Hong Kong Chongqing International Institute for Immunology Hong Kong China; ^5^ Chinese Academy of Medical Sciences and Peking Union Medical College Institute of Dermatology Nanjing China; ^6^ Key Laboratory of Basic and Translational Research on Immune‐Mediated Skin Diseases Nanjing China; ^7^ Chinese Academy of Medical Sciences Jiangsu Key Laboratory of Molecular Biology for Skin Diseases and STIs Nanjing China

**Keywords:** AIM2, systemic lupus erythematosus (SLE), T follicular helper cells (T_FH_)

## Abstract

Systemic lupus erythematosus (SLE) is a chronic autoimmune disease that involves T follicular helper (T_FH_) cell‐mediated humoral immune responses. Absent in melanoma 2 (human AIM2 and murine Aim2) is a well‐known component of the inflammasome in the innate immune system. Surprisingly, we observed that in SLE patients, upregulated levels of AIM2 expression were found in peripheral blood and skin lesions, with the highest levels detected in T_FH_‐like cells. In the *CD4^cre^Aim2^fl/fl^* conditional knockout mice, a markedly reduced T_FH_ cell response was observed, with significantly lower levels of serum autoantibodies and proteinuria, as well as profoundly reduced renal IgG deposition in pristane‐induced lupus mice. Mechanistically, IL‐21 was found to recruit hydroxymethyltransferase ten‐eleven translocation 2 (TET2) to the *AIM*2 promoter, resulting in DNA demethylation and increased transcription of AIM2. In addition, AIM2 could regulate c‐MAF expression to enhance IL‐21 production, which consequently promoted T_FH_ cell differentiation. Our results have identified a role of AIM2 in promoting the T_FH_ cell response and further revealed that the IL‐21‐TET2‐AIM2‐c‐MAF signalling pathway is dysregulated in lupus pathogenesis, which provides a potential therapeutic target for SLE.

## Graphical abstract

- Absent in melanoma 2 (AIM2) is a component of AIM2 inflammasome and links with innate immunity in SLE.
- Enriched AIM2 level has been found in T_FH_‐like cells, in circulated environment and skin lesions in lupus patients.
- AIM2 affects SLE development by regulating T_FH_ cell differentiation, which is regulated by IL‐21‐mediating TET2 recruitment and interaction between AIM2 and c‐MAF in T_FH_ signalling pathways.

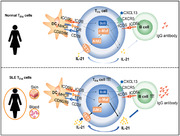

## 1 INTRODUCTION

As a systemic autoimmune disease, systemic lupus erythematosus (SLE) affects multiple organs and has a salient feature of abundant autoantibodies against self‐nuclear materials.^1^ The oetiology of SLE remains unclear, but earlier research has revealed a pivotal function of CD4^+^ T cells in lupus pathogenesis.^2^ During the last decade, it became clear that follicular helper T (T_FH_) cells promote the overactivation of immune complexes and the response of autoimmune T and B cells, exerting critical pathogenic effects on SLE. Additionally, increased T_FH_ cells have also been found in SLE mouse models, where T_FH_ cells are required for lupus‐like pathology.^3^ Maturation of the germinal centre (GC) indispensably relies on T_FH_ cells, which commit themselves with the help of a group of ‘T_FH_ molecules’, including interleukin‐4 (IL‐4), IL‐21, B‐cell lymphoma 6 (BCL6), C‐X‐C chemokine receptor type 5 (CXCR5), inducible T cell co‐stimulator (ICOS), CD40 ligand (CD40L), signal transducer and activator of transcription 3 (STAT3) and c‐musculoaponeurotic fibrosarcoma (c‐MAF), during T_FH_ cell development.^4^, ^5^, ^6^ As a critical downstream transcription factor in ICOS regulation in T_FH_ cell development, c‐MAF activates IL‐21 promoters and enhancers, consequently regulating and sustaining the production of IL‐21.^7^ Except for human findings, a previous study has suggested that murphy roths large/lymphoproliferative (MRL/lpr) lupus mice experienced reduced autoantibody levels and ameliorated clinical symptoms by impairing c‐MAF regulation in T_FH_ cells after dexamethasone treatment.^8^ Although T_FH_ cells largely contribute to autoimmune pathogenesis, further exploration is needed to identify new molecular regulators involved in T_FH_ cell differentiation and determine how these regulatory mechanisms contribute to lupus development.^9^

Absence in melanoma 2 (human AIM2 and murine Aim2) is a DNA sensor that assembles the AIM2 inflammasome to defend against pathogen‐associated molecular patterns (PAMPs) in innate immunity. Recent studies have mainly focused on the inflammasome‐dependent role of AIM2 in innate defense. However, emerging evidence suggests a critical function for AIM2 in adaptive immunity, including in tumours and autoimmune diseases.^10^, ^11^, ^12^, ^13^ Several studies have shown that Aim2 restrains clinical symptoms in experimental autoimmune encephalomyelitis (EAE) mice by stabilising T regulatory (Treg) cells,^12^, ^14^ suggesting that inflammasome‐independent Aim2 participates in the regulation of the CD4^+^ T‐cell response in autoimmune models. However, the role of AIM2 in the progression of SLE remains obscure. Previous evidence has indicated that AIM2 in innate immune cells is closely related to disease severity in SLE patients and facilitates mouse disease symptoms in an apoptotic DNA‐induced lupus model.^15^ In contrast, newly published data have identified a protective role of AIM2 in innate immune cells during SLE development by suppressing the expression of type I interferon (IFN)‐induced genes.^16^ To date, it is still not known whether AIM2 is involved in the regulation of the T_FH_ cell response and thereby contributes to the pathogenesis of SLE.

Epigenetic dysregulation is well recognised in the pathogenesis of SLE.^17^, ^18^ Lines of evidence have demonstrated that epigenetic modifications, in concert with the regulation of transcription factors, contribute to the differentiation and function of CD4^+^ helper T cells.^19^ Generally, DNA methylation represses gene expression, whereas DNA demethylation/hydroxymethylation contributes to the reactivation of gene transcription. Furthermore, transcription factors directly regulate DNA methylation by binding to target gene loci.^20^ For T_FH_ cells, BCL6 binding to specific DNA sequences reduces the recruitment and translocation of hydroxymethyltransferase ten‐eleven translocation 1 (TET1), which decreases the level of 5‐hydroxymethylcytosine (5hmC).^21^ IL‐21 can enhance the recruitment of the hydroxymethyltransferase TET2 in the promoter region of *BCL6* in lupus patients.^5^ Since reduced DNA methylation in the *AIM2* promoter in CD4^+^ T cells has been observed in lupus patients,^22^, ^23^ we wondered whether DNA methylation‐modulated AIM2 might regulate T_FH_ cell differentiation, which can exert pathogenic effects on SLE.

To test our hypothesis, AIM2 expression was first explored in patients with SLE. Notably, we detected enhanced AIM2 expression in both normal tonsil T_FH_ cells and T_FH_‐like cells in the peripheral blood and skin lesions of SLE patients. Furthermore, to define how AIM2 modulates T_FH_ cell function, we generated conditional knockout (CKO) mice with an AIM2 deficiency in CD4^+^ T cells. During lupus development induced upon pristane administration, these mice showed a markedly reduced T_FH_ response and serum autoantibody levels, together with profoundly reduced renal immunoglobulin G (IgG) deposition. Mechanistically, AIM2 promoted T_FH_ cell differentiation via the c‐MAF signalling pathway, while IL‐21 was found to promote the expression of AIM2 by increasing TET2 enrichment in the *AIM2* promoter region. Together, these results identify a previously unrecognised DNA methylation‐related modulation mechanism underlying the regulation of AIM2 in the T_FH_ cell response and SLE progression.

## 2 MATERIALS AND METHODS

### 2.1 Human sample collection

Normal controls (NCs) were matched to the patients by age, sex and ethnicity. All blood samples and skin and tonsil biopsies of patients were collected from the Second Xiangya Hospital. Blood samples and skin biopsies of NCs were collected from the Changsha Blood Center and the Second Xiangya Hospital, respectively. The systemic lupus erythematosus disease activity index (SLEDAI) score was used for the assessment of disease activity in SLE patients.^24^ More detailed information can be found in Tables S1–S6. The ethics permit for humans and mice was obtained from the Institutional Committee of Ethics at the Second Xiangya Hospital (No. 2019‐30).

### 2.2 Mice

*Aim2^‐/‐^*, C57BL/6J and B6D2F1 mice were obtained from the Jackson Laboratory, the Slack Company and the Beijing Vital River Laboratory Animal Technology Company, respectively. The *CD4^cre^Tet2^fl/fl^* mice were gifts from Dr. Yoshimura Aki. The design and generation of *CD4^cre^Aim2^fl/fl^* mice were performed by Shanghai Biomodel Organism Science & Technology Development Company. *Aim2^fl/fl^* mice were generated on the C57BL/6 genetic background. Briefly, *Aim2^fl/fl^* mice were first generated by using a donor vector which contained loxP sites flanking exon 9 of *Aim2* (ENSMUST00000147604.7). *CD4^cre^Aim2^fl/fl^* mice were then generated by crossing *Aim2^fl/fl^* mice with mice expressing Cre recombinase from the CD4 T‐cell promoter (CD4^cre^). Genotypes of the *Aim2^fl/fl^* mice were confirmed by performing polymerase chain reaction (PCR) analyses of genomic DNA isolated from mouse ears and by using the following primers: forward 5′‐GGGTGGGATGAGATGAGAGTGAGC‐3′, reverse 5′‐GGGTGGGATGAGATGAGAGTGAGC‐3′. Animal care and experimental procedures followed the normalised guidelines in China.

### 2.3 Cell differentiation and proliferation assays in culture

#### 2.3.1 Human

A total of 2.5 × 105 naïve CD4+ T cells were seeded in anti‐CD3 antibody‐precoated plates (Calbiochem, 5 μg/ml) and further treated with anti‐CD28 antibody (Calbiochem, 2 μg/ml) and different cytokines (PeproTech) as follows. T_FH_ cells: IL‐6 (20 ng/ml), transforming growth factor‐β (TGF‐β) (5 ng/ml), IL‐12 (10 ng/ml) and IL‐21 (20 ng/ml); TH1 cells: IL‐2 (10 ng/ml), IL‐12 (10 ng/ml) and anti‐human IL‐4 antibody (10 ng/ml); TH2 cells: IL‐2 (10 ng/ml), IL‐4 (25 ng/ml) and anti‐human IFN‐γ antibody (10 ng/ml); TH17 cells: IL‐1β (12.5 ng/ml), IL‐6 (25 ng/ml), IL‐21 (25 ng/ml), IL‐23 (10 ng/ml), TGF‐β (10 ng/ml), anti‐human IL‐4 antibody (5 ng/ml) and anti‐human IFN‐γ antibody (5 ng/ml); Treg cells: IL‐2 (10 ng/ml) and TGF‐β (5 ng/ml).

#### 2.3.2 Mouse

Naïve CD4^+^ T cells (5 × 10^5^) were seeded in anti‐CD3 antibody‐precoated plates (BD Bioscience, 2 μg/ml). A mixture of anti‐CD28 antibody (BD Bioscience, 1 μg/ml), IL‐6 (10 ng/ml), IL‐21 (10 ng/ml), anti‐IL‐4 antibody (10 ng/ml) and anti‐IFN‐γ antibody (10 ng/ml) was added to the culture with cells for 3 days for T_FH_ cell differentiation. For the cell proliferation assay, 5 × 10^5^ CD4^+^ T cells stained with carboxyfluorescein succinimidyl ester (CFSE, BD Bioscience) underwent treatment with anti‐CD3 and anti‐CD28 antibodies as described above for 5 days.

### 2.4 Antisense oligonucleotide transfections

The Amaxa Nucleofector transfection system (Lonza) was used in the human T_FH_ differentiation assay. Briefly, 2.5 μl antisense oligonucleotide (ASO) (20 μM) was added to a mixture of resuspended cells and 100 μl nucleofector solution. Electrotransfection was carried out on an Amaxa Nucleofector apparatus (Lonza) and cultured in RPMI 1640 complete medium (Gibco). Cell culture media were refreshed, and T_FH_ cell cytokine stimuli were added 6 h after transfection. Five days later, T_FH_ cells were collected and prepared for following real time‐PCR. The sequence of the AIM2 ASO (RiboBio, lnc6200529012211) is shown in Table S8.

### 2.5 Lupus mouse models

A pristane‐induced model was generated as previously described.^25^ *CD4^cre^Aim2^fl/fl^* and *Aim2^fl/fl^* mice were injected with 500 μl pristane (Sigma–Aldrich) intraperitoneally. Chronic graft versus host disease (cGVHD) lupus model was reported in a previous study.^26^ A total of 5 × 10^7^ CD8^+^ T‐cell‐depleted lymphocytes from female *Aim2^‐/‐^* or wild‐type (WT) mice were transferred to each B6D2F1 mouse intravenously via the tail vein. Colorimetric assay strips (URIT) were used for proteinuria assessment. Serum and mouse kidney biopsies were collected when mice were sacrificed. Assessments of anti‐double strained DNA (anti‐dsDNA) antibodies and anti‐nuclear antibodies (ANA) in diluted serum (1:100) were performed by using ELISA kits (Alpha Diagnostic).

### 2.6 Keyhole limpet haemocyanin mouse model

To generate a keyhole limpet haemocyanin (KLH) mouse model, 200 μg complete Freund's adjuvant （CFA）‐emulsified KLH (Sigma–Aldrich) was subcutaneously injected into the tail and neck of the mice.^27^ 4‐hydroxy‐3‐nitrophenyl acetyl‐KLH (NP‐KLH,Sigma–Aldrich) was used to explore the affinity maturation of NP‐specific antibodies. Serum was collected and seeded on 10 mg/ml 4‐hydroxy‐3‐nitrophenyl acetyl‐bovine serum albumin (NP‐BSA)‐precoated plates (Bioresearch Technologies). The antibodies on the plates were then detected via horseradish peroxidase (HRP) goat anti‐mouse IgG and IgM (Southern Biotech). The absorbance at 450 nm was read by a multimode plate reader (Perkin Elmer).

### 2.7 Flow cytometry and confocal microscopy

Cells were incubated with antibodies in the cold (30 min). To stain intracellular markers or cytokines, the Human Foxp3 Buffer Set (BD Biosciences) was used. For AIM2 staining, fluorescence minus one control (FMO), representing no AIM2 expression, was used for normalisation of the flow cytometric results. Cells were acquired by CyTek Aurora, and data were analysed by FlowJo 10.4. Cells for confocal microscopy were finally analysed by confocal microscopy (Zeiss LSM 780). The antibodies used in flow cytometry and confocal microscopy are listed in Table S6.

### 2.8 Histology and immunohistochemistry staining

Sections (4 mm) from mouse lymph nodes and kidneys were treated with formalin and paraffin and further subjected to either haematoxylin and eosin (H&E) staining or immunohistochemistry (IHC) staining. For the latter, sections were further dewaxed with xylene, 95% ethanol and 70% ethanol. Primary and secondary antibodies were then sequentially applied at room temperature (RT) (30 min). The antibodies are listed in Table S6. Slides were analysed by a digital fluorescence microscope (Leica) and NLS Elements Basic Research Imaging Software (Leica). For multicolour IHC staining, an Opal Seven‐Color Kit (Perkin Elmer) was applied. Briefly, sections of human skin and tonsil tissues underwent epitope retrieval in citrate buffer (Perkin Elmer) and incubation with blocking buffer (Perkin Elmer) and then primary antibodies at RT (30 min). Background staining was further reduced, and sections were rinsed in citrate buffer. Sections were finally scanned by a Vectra imaging system (Perkin Elmer, Vectra 3.0.3) and analysed by inForm (Perkin Elmer, inForm 2.3.1).

### 2.9 Chromatin immunoprecipitation

A total of 3 × 10^6^ cells were crosslinked in 1% formaldehyde, rinsed in cold Phosphate‐buffered saline (PBS), lysed in radioimmunoprecipitation assay buffer (RIPA buffer), sonicated to shear the DNA and sedimented. Quantified supernatants were immunoprecipitated with the anti‐Tet2 antibody (Santa Cruz) on ice overnight. A chromatin immunoprecipitation (ChIP) Assay Kit (Millipore) was then used, and the amount of immunoprecipitated *AIM2* DNA was assessed by real‐time PCR. The primer sequences (BioSune Biotechnology) can be found in Table S8.

### 2.10 Bisulphite genomic sequencing

CD4^+^ T cells (5 × 10^6^) from SLE patients and normal healthy human, and naïve CD4^+^ T cells (5 × 10^6^) from normal healthy human were isolated by using Miltenyi beads. T_FH_ cells (5 × 10^6^) were differentiated by using the stimulus method mentioned above. Sodium bisulphite (EZ DNA Methylation kit) was used for bisulphite conversion of DNA, and then the AIM2 promoter fragment was amplified using nested primers. The fragments were cloned (pGEM‐T Easy Vector Systems), and 10 independent clones were sequenced by BioSune Biotechnology Company. Nest primers for AIM2 were purchased from BioSune Biotechnology Company (Table S8).

### 2.11 Western blotting

In the Western blotting assay, to detect the nuclear and cytosolic localisation of the murine Aim2 protein, a cytoplasmic and nuclear extraction kit (Invent) was used. Stripping solution was used for blots for removal of the antibodies from the membrane when the detected proteins had similar molecular weights. More information about the antibodies used is shown in Table S7.

### 2.12 Co‐immunoprecipitation

Live cells were lysed in RIPA buffer. Dynabeads Protein G Immunoprecipitation Kit (Invitrogen) was then used in the following co‐immunoprecipitation (Co‐IP) assays. Briefly, the mix of cell protein and antibody was incubated on a magnet. Proteins that are not immobilised on beads can be removed by rinsing the protein mix, while the bait–protein complexes can be generated. After incubation, Western blotting was used for the final detection of whether the target protein was present or absent. The following antibodies for Western blotting were used: anti‐c‐MAF (Abcam, ab230928, 1:1000) and anti‐human AIM2 (Abcam, ab204995, 1:1000) antibodies.

### 2.13 Predicted binding domains

To identify potential functional domains contributed to the interactions between AIM2 and c‐MAF, silico approaches were conducted， and structural fragments of AIM2 (e.g., 3RN5) and MAF had been crystallised. Firstly, to model protein structures, individual full‐length amino acid sequences of AIM2 and MAF were used as inputs of the I‐TASSER server. In further analysis, we then used the ResProx server for selecting potential models. Next, to estimate potential structural interfaces in the interaction of AIM2‐c‐MAF, Cluspro V2.0 was used. Protein interaction databases, interaction models matched with the literature and structural constraints were screened and the final ones with the highest structural scores were got. Visualisation of structure was prepared using PyMOL V2.1. AIM2 and c‐MAF were found in the nucleus and cytoplasm, estimation of nuclear localisation signals in the full sequences of AIM2 and c‐MAF was conducted using the online server cNLS Mapper.

### 2.14 RNA‐seq and bioinformatics analysis

Human naïve T cells, T_FH_ cells and murine splenic CD4^+^ T cells were collected for RNA extraction, sample detection, enrichment, amplification, library preparation and Illumina sequencing by the SHBIO Company. All RNA‐seq data were analysed by R Studio. To identify differentially expressed genes, the R package limma was used. The package cluster Profiler was used for Gene Ontology enrichment analysis. A *p*‐value <0.05 and fold‐change values >1.5‐fold were used in the human analyses, while fold‐change values >1.2‐fold were used in the murine analysis.

### 2.15 Statistical methods

Statistics were calculated by one‐way analysis of variance (ANOVA) in the comparison of multiple groups and by unpaired two‐tailed *t*‐test in the comparison of two groups in SPSS software (18.0). *p < .05, **p < .01, ***p < .001, ****p < .0001.

## 3 RESULTS

### 3.1 Increased AIM2 expression in human T_FH_ cells and in vitro differentiated T_FH_ cells

**FIGURE 1:**
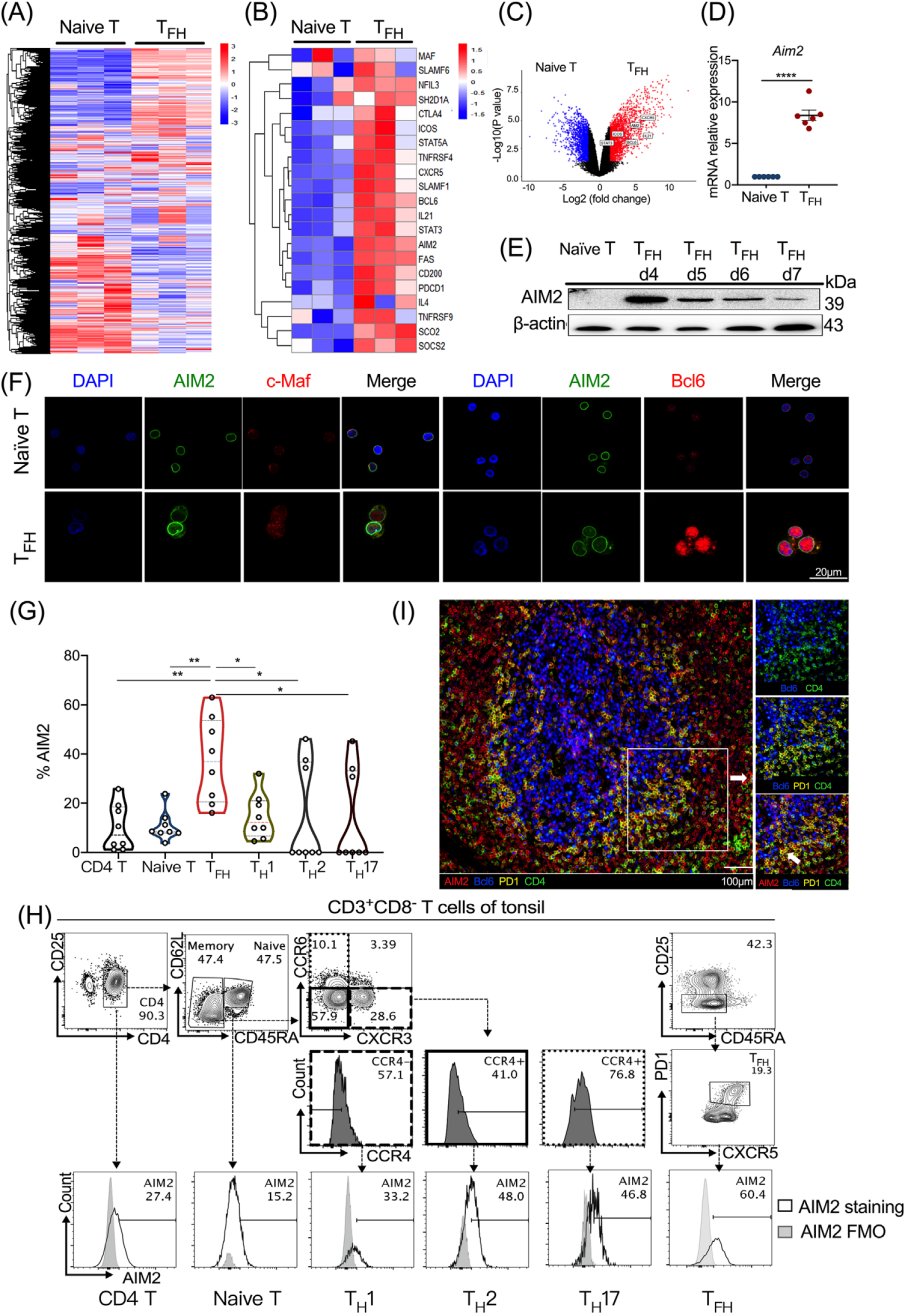
Absent in melanoma 2 (AIM2) is highly expressed in human T follicular helper (T_FH_) cells and in vitro differentiated T_FH_ cells. (A–F) Naïve CD4^+^ T cells from the peripheral blood of healthy controls were used for the differentiation of T_FH_ cells in vitro for 5 days (*n* = 10, detailed information of the donors is shown in Table S6). Heatmaps of mRNA‐seq analysis of (A) global genes and (B) T_FH_ cell signature genes, as well as (C) volcano plots of upregulated (red) and downregulated (blue) genes, are shown. (D) Real‐time PCR was performed to detect the *Aim2* mRNA levels of naïve CD4^+^ T cells and T_FH_ cells (d5) (*n* = 6). (E) AIM2 accumulation during T_FH_ cell differentiation is shown. (F) Representative images of AIM2, c‐Maf and Bcl6 in naïve CD4^+^ T cells and T_FH_ cells as determined by confocal microscopy. (G) Data plots of AIM2 expression in CD4^+^ T‐cell subsets from human tonsils by flow cytometry (*n* = 8, detailed information of the donors is shown in Table S2). (H) Gating strategy and representative flow cytometric plots of AIM2 percentages among various CD4^+^ T‐cell subsets from human tonsils in (G). (I) Representative images of AIM2^+^ T_FH_ cells from human tonsils by multicolour immunohistochemistry staining. Bars show the mean ± SEM. *p < .05, **p < .01, ***p < .001, ****p < .0001

To investigate the underlying molecular basis of T_FH_ cells in the pathogenesis of SLE, we performed mRNA‐seq assays of T_FH_ cells during cell differentiation in vitro and compared the transcriptional profiles between naïve CD4^+^ T and T_FH_ cells in culture. Notably, we found a drastic increase in AIM2, together with T_FH_ signature genes such as *BCL6*, *IL21*, *CXCR5* and *PD1*, during T_FH_ cell differentiation (Figure 1A–C). Real‐time PCR analysis revealed an approximately ninefold AIM2 enrichment in T_FH_ cells in contrast with naïve CD4^+^ T cells (Figure 1D). Except for transcriptomic expression of AIM2, we also collected T_FH_ cells along in vitro differentiation and performed proteinic level assessment. Immunoblotting results then showed that AIM2 increased with T_FH_ cell differentiation but then declined gradually (Figure 1E). To support this notion, higher expression levels of AIM2 together with T_FH_ transcription factors such as BCL6 and c‐MAF were also observed in T_FH_ cells by confocal microscopy (Figure 1F). Unexpectedly, T_FH_‐AIM2 resided as a condensed circle positioned adjacent to or in the nuclear membrane of T_FH_ cells, which suggested that AIM2 may locate in nucleus and have a function relating to transcriptional programs in adaptive immunity.

T_FH_ cells mainly reside in lymphoid organs. To further confirm our findings in vitro system, we examined AIM2 expression levels in human tonsil tissues. After normalisation of the FMO control, flow cytometric results revealed that tonsil T_FH_ cells exhibited the highest levels of AIM2 among the CD4^+^ T‐cell subsets (Figure 1G and H). Similarly, T_FH_ cells had the highest AIM2 expression among various helper CD4^+^ T‐cell subsets in culture (Figure S1A and B). Moreover, multicolour IHC staining results identified that AIM2 was widely expressed in human tonsils (Figure 1I) and highly expressed in CD4^+^Bcl6^+^PD1^+^ T_FH_ cells (Figure S1C). The T_FH_ cells, represented by CD4^+^Bcl6^+^PD1^+^ cells, overlapped with the area of AIM2^+^ cells (Figure 1I), and the follicular mantle zone exhibited intense AIM2 expression (Figure S1D and E). Collectively, these findings supported a crucial biological role of AIM2 in T_FH_ cells.

### 3.2 Increased AIM2 in T_FH_‐like cells from peripheral blood and skin lesions of lupus patients

**FIGURE 2:**
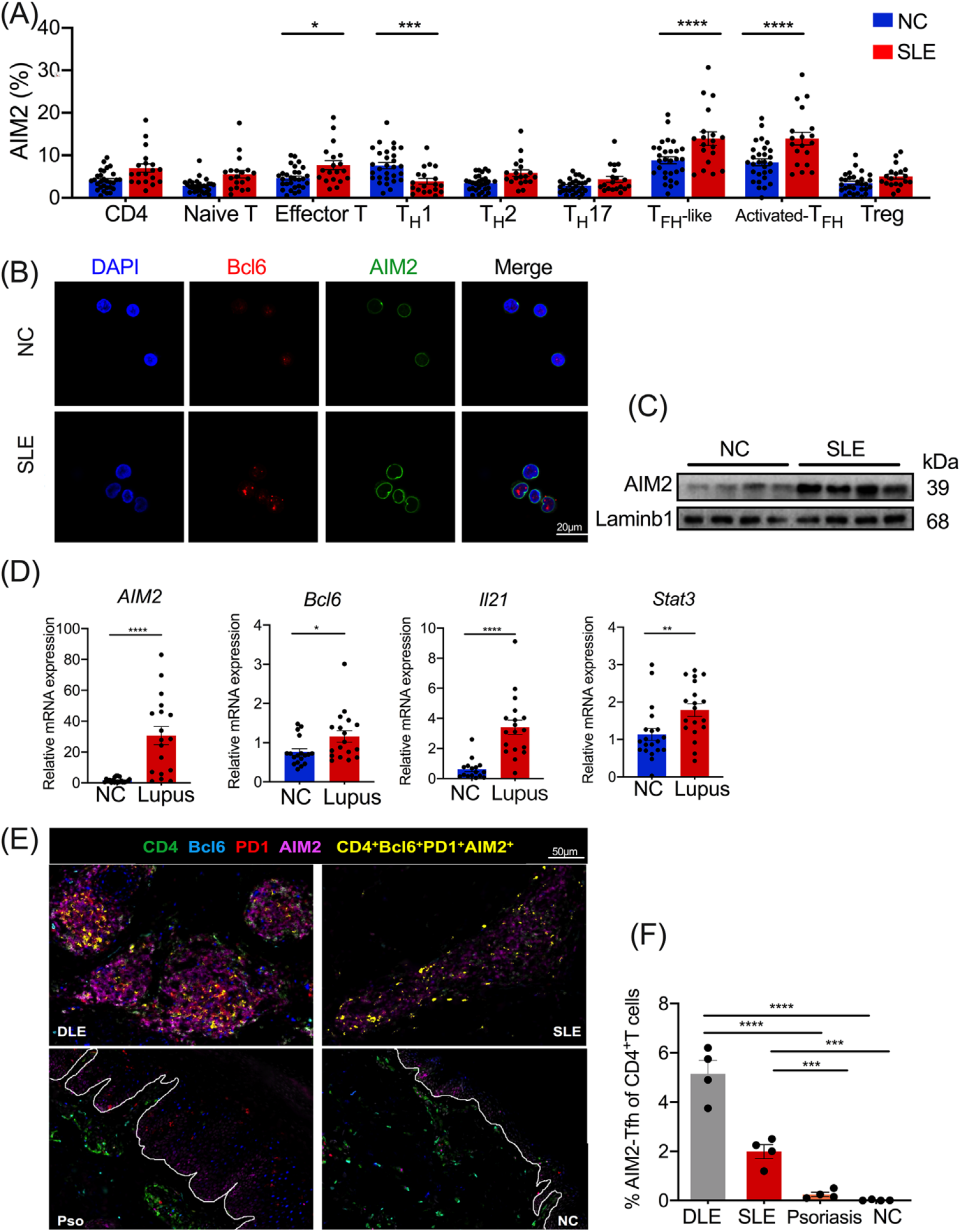
Absent in melanoma 2 (AIM2) is highly expressed in T follicular helper (T_FH_)‐like cells from peripheral blood and skin lesions in systemic lupus erythematosus (SLE) patients. (A) AIM2 expression in CD4^+^ T‐cell subsets in peripheral blood from normal controls and SLE patients was assessed by flow cytometry (*n* = 19 or 29). (B) Detection of AIM2 and Bcl6 in peripheral CD4^+^ T cells from normal controls and SLE patients was performed by confocal microscopy (*n* = 3). (C) AIM2 accumulation in peripheral CD4^+^ T cells from SLE patients and normal controls is shown (*n* = 4). (A–C) Detailed information of the donors is shown in Table S3. (D) Real‐time PCR was performed to assess the mRNA levels of *Aim2* and T_FH_‐related genes in skin lesions from lupus patients and normal controls (*n* = 19). (E) Representative images of AIM2 expression in skin lesion T_FH_‐like cells (CD4^+^Bcl6^+^PD1^+^) from patients with discoid lupus erythematosus (DLE), SLE, or psoriasis and normal controls determined by multicolour immunochemistry staining (*n* = 4). (F) Quantified percentage of AIM2^+^ T_FH_ cells in (E). (D–F) Detailed information of the donors is shown in Tables S1 and S4. Bars show the mean ± SEM. *p < .05, **p < .01, ***p < .001, ****p < .0001

T_FH_ cells promote the development of SLE, therefore, we suspect AIM2 may also affect SLE pathology in T_FH_ cell signalling pathway. To shed light on the potential function of AIM2 in SLE, we first detected AIM2 levels in various subsets of CD4^+^ T cells in the peripheral blood of SLE patients and NCs by flow cytometry. Generally, AIM2 levels were higher in most circulating CD4^+^ T subsets (except for T_H_1 cells) from SLE patients (Figures 2A and S2A). However, T_FH_‐like cells, along with activated T_FH_‐like cells, expressed the highest AIM2 levels in SLE patients among all other T‐cell subsets (Figure 2A). These findings demonstrated a potential pathogenic function of CD4^+^ T cell‐AIM2 in SLE, which might be mediated by T_FH_ cell signalling pathways.

We then detected a higher AIM2 accumulation in SLE patients than in NCs by confocal microscopy and Western blot analysis (Figure 2B and C). In skin lesions, higher mRNA levels of *AIM2* and T_FH_‐related genes, including *BCL6*, *IL21* and *STAT3*, were found in lupus patients than in NCs (Figure 2D). Remarkably, only lupus patients but not psoriasis patients or NCs had high AIM2 expression among infiltrating CD4^+^ T cells in skin lesions (Figure S2B–D), which suggested highly expressed AIM2 may contribute to lupus pathology. To further assess the function of T_FH_‐AIM2 in lupus, we examined the co‐localisation of T_FH_‐like cells and AIM2 in patients with discoid lupus erythematosus (DLE), SLE, psoriasis and NCs. Lupus patients including DLE and SLE, exhibited significantly higher frequencies of AIM2^+^ T_FH_‐like cells than psoriasis patients and NCs (Figure 2E and F). Accordingly, we also observed that the cells with the highest AIM2 levels were AIM2^+^ T_FH_‐like cells (CD4^+^PD1^+^Bcl6^+^AIM2^+^) in lupus patients (Figure S2E). Up to this point, our findings revealed a potential function of AIM2‐mediated T_FH_ cells in lupus pathogenesis.

### 3.3 AIM2 deficiency impairs CD4^+^ T‐cell differentiation

To test the influence of AIM2 in regulating the T‐cell response in vivo, we generated CKO *CD4^cre^Aim2^fl/fl^* mice (Figure S3B), and flow cytometric analysis demonstrated that deficiency of Aim2 in CD4^+^ T cells did not alter the frequencies of T and B cells in draining lymph nodes (dLNs) or thymic T‐cell development (Figure S3C and D). In comparison with the *Aim2^fl/fl^* control mice, the *CD4^cre^Aim2^fl/fl^* mice exhibited decreased CD4^+^ T cells and increased CD8^+^ T cells in dLNs (Figure S3E), indicating the involvement of CD4^+^ T cell‐AIM2 in CD4^+^ T‐cell differentiation and expansion. Importantly, we did not detect altered frequencies of naïve CD4^+^ T or effector CD4^+^ T cells between *CD4^cre^Aim2^fl/fl^* mice and control mice at steady state (Figure S3F). However, after treatment with anti‐CD3 and anti‐CD28 antibodies in culture, *CD4^cre^Aim2^fl/fl^* mice had lower effector CD4^+^ T and higher naïve CD4^+^ T‐cell frequencies than control mice (Figure S3G). We then analysed CD4^+^ T‐cell proliferation by assessing CFSE fluorescence after stimulation with anti‐CD3 and anti‐CD28 antibodies in vitro. In *Aim2^fl/fl^* mice, there were approximately 30% more proliferated CFSE^+^CD4^+^ T cells than in the CKO mice (Figure S4H). Altogether, these findings supported the idea that AIM2/Aim2 regulates CD4^+^ T‐cell expansion in secondary lymphoid tissues, possibly in a T‐cell receptor (TCR) signalling pathway.

### 3.4 CD4^+^ T‐cell‐intrinsic AIM2 deficiency decreases the antigen‐specific T_FH_ response

**FIGURE 3:**
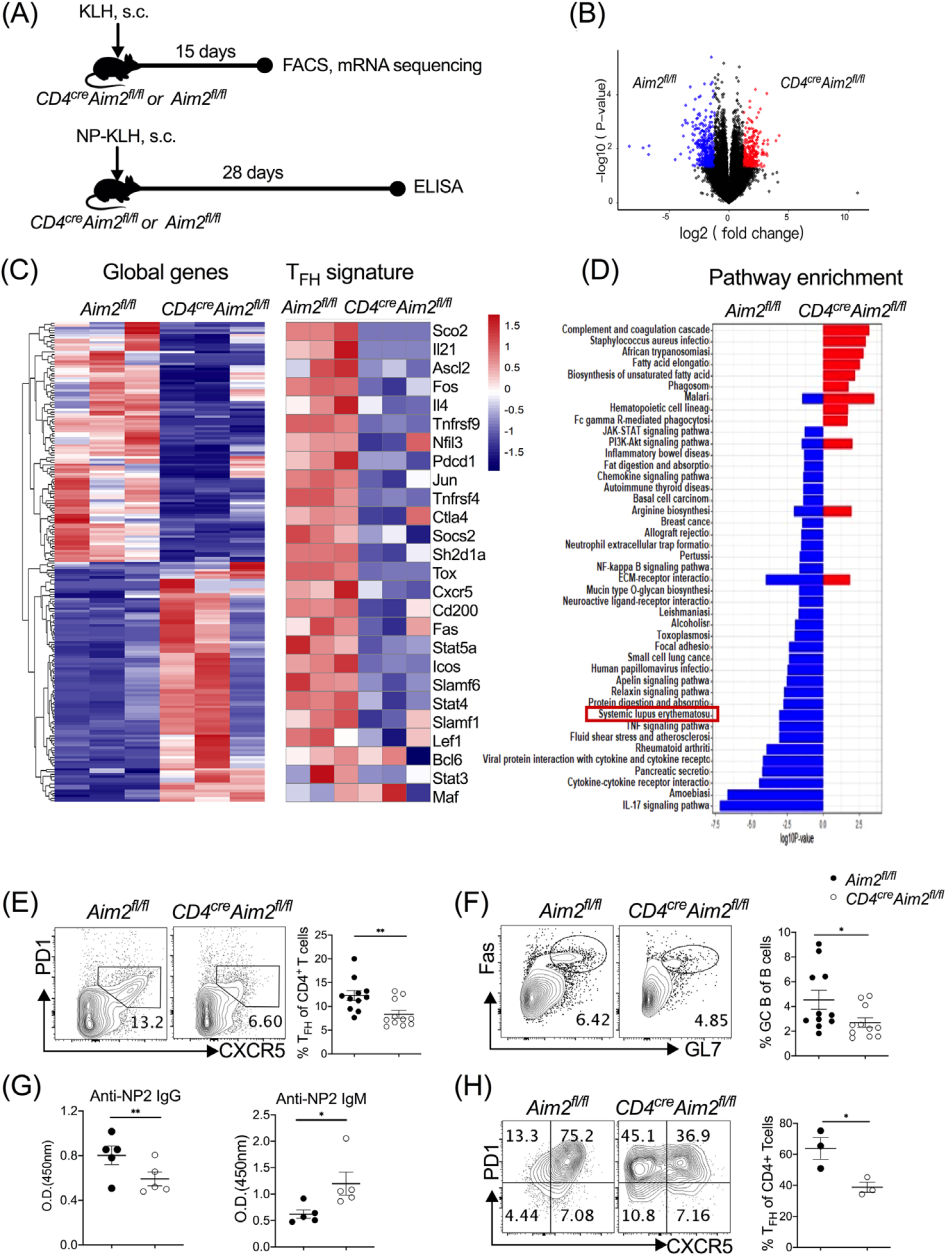
Deficiency of CD4^+^ T‐cell‐intrinsic absent in melanoma 2 (AIM2) impairs T follicular helper (T_FH_) cell differentiation and IgG production after NP‐keyhole limpet haemocyanin (KLH) immunisation. (A) Schematic of the KLH and NP‐KLH mouse model. (B–F) *Aim2^fl/fl^* and *CD4^cre^Aim2^fl/fl^* mice were challenged with KLH for 15 days (*n* = 11). (B) Volcano plot of mRNA‐seq analysis of CD4^+^ T cells from *Aim2^fl/fl^* and *CD4^cre^Aim2^fl/fl^* mice. Upregulated (red) and downregulated (blue) genes are shown (*n* = 3). (C) Heatmaps of global gene expression and T_FH_ cell signature gene expression in CD4^+^ T cells from *Aim2^fl/fl^* and *CD4^cre^Aim2^fl/fl^* mice by mRNA‐seq. (D) The pathway enrichment analysis of RNA‐seq. Representative flow cytometric profiles and data plots of (E) T_FH_ (CD4^+^CXCR5^+^PD1^+^) cells and (F) germinal centre (GC) B (B220^+^GL7^+^Fas^+^) cells in draining lymph nodes (dLNs) (*n* = 11). (G) Serum levels of anti‐NP IgG and IgM in *Aim2^fl/fl^* and *CD4^cre^Aim2^fl/fl^* mice challenged with NP‐KLH for 28 days (*n* = 5). (H) Naïve CD4^+^ T cells from *Aim2^fl/fl^* and *CD4^cre^Aim2^fl/fl^* mice were used for in vitro T_FH_ cell differentiation for 3 days. Representative flow cytometric profiles and data plots of T_FH_ cells (CD4^+^CXCR5^+^PD1^+^) are shown (*n* = 3). Bars show the mean ± SEM. *p < .05, **p < .01

KLH is an effective protein antigen and widely used in immunisation of mouse models in vivo.^27^ To address the function of Aim2 in T_FH_ cell differentiation in vivo, KLH was injected into CKO mice (Figure 3A). The mRNA‐seq results first demonstrated completely different gene expression signatures between CKO and control mice after KLH challenge (Figure 3B and C). Notably, we found significantly reduced expression of T_FH_‐related genes, including *IL4*, *IL21*, *PD1*, *CXCR5*, *ICOS*, *STAT4* and *ASCL2*, in *CD4^cre^Aim2^fl/fl^* mice (Figure 3C). Pathway enrichment analysis revealed upregulated enrichment of the SLE pathway in *Aim2^fl/fl^* mice but not in *CD4^cre^Aim2^fl/fl^* mice (Figure 3D), suggesting that CD4^+^ T‐cell‐intrinsic Aim2 may promote SLE development. Flow cytometric results further identified that lack of CD4^+^ T‐cell‐intrinsic Aim2 resulted in lower frequencies of T_FH_ cells and GC B cells, which were also consistent with the sequencing data (Figure 3E and F). T_FH_ cells are crucial for GC B‐cell development and antibody production. To explore AIM2 in the regulation of KLH‐specific antibody responses, we injected CKO mice with NP‐KLH. Correspondingly, lower serum KLH‐specific IgG antibodies (anti‐NP2 IgG) were found in *CD4^cre^Aim2^fl/fl^* mice, however, *CD4^cre^Aim2^fl/fl^* mice experienced increased KLH‐specific IgM antibodies (anti‐NP2 IgM) (Figure 3G). IgM antibodies often correlate with protection from organ damage and are regarded as protective markers in SLE pathogenesis,^28^ which may explain the discrepancy of KLH‐specific antibody production between IgG and IgM. Next, we aimed to validate Aim2 regulation in the T_FH_ cell differentiation in vitro system. Similar to the in vivo results, fewer CD4^+^CXCR5^+^PD1^+^ cells were observed in *CD4^cre^Aim2^fl/fl^* mice (Figure 3H), suggesting that depletion of CD4^+^ T‐cell‐intrinsic Aim2 impairs the in vivo differentiation of T_FH_ cells.

### 3.5 AIM2 deficiency in CD4^+^ T cells ameliorates lupus development in mice

**FIGURE 4:**
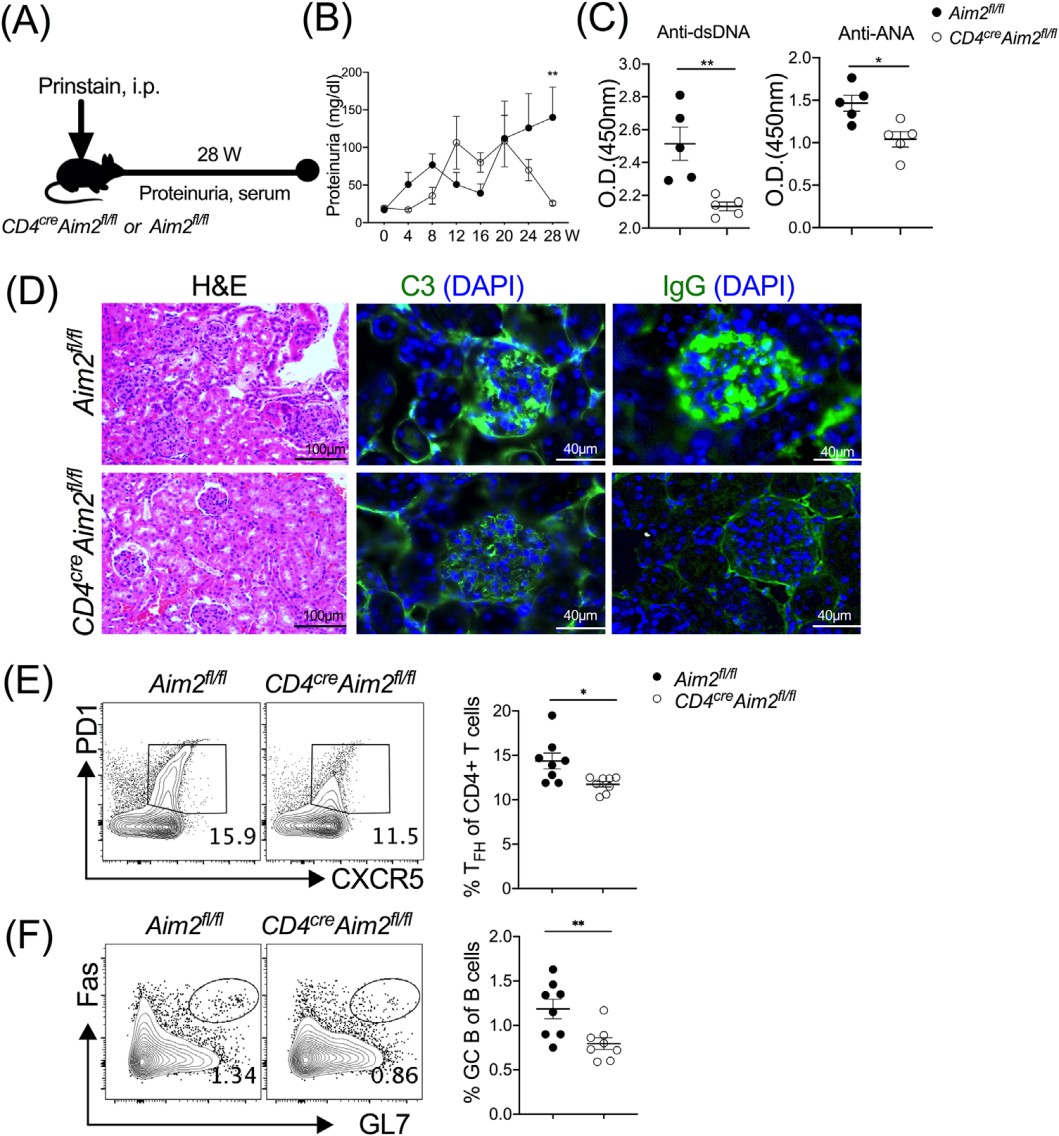
Absent in melanoma 2 (AIM2) deficiency in CD4^+^ T cells ameliorates lupus development in mice. A pristane‐induced lupus mouse model was generated in *Aim2^fl/fl^* and *CD4^cre^Aim2^fl/fl^* mice. (A) Schematic of pristane‐induced lupus model. (B) Proteinuria and (C) serum levels of anti‐dsDNA antibody and ANA in *Aim2^fl/fl^* and *CD4^cre^Aim2^fl/fl^* mice were assessed (*n* = 5). (D) Representative images of haematoxylin and eosin (H&E)‐stained kidneys and immunofluorescence staining with anti‐C3 and anti‐IgG antibodies in the kidney glomeruli are shown. Representative profiles of flow cytometric and data plots of (E) T follicular helper (T_FH_) (CXCR5^+^PD1^+^CD4^+^) cells and (F) germinal centre (GC) B (GL7^+^Fas^+^B220^+^) cells (*n* = 8). Bars show the mean ± SEM. *p < .05, **p < .01

Pristane lupus model is the most common SLE mouse model, characterised with SLE‐like symptoms including severe nephritis, proteinuria and increased autoantibody levels.^25^ To examine how CD4^+^ T cell‐Aim2 affects SLE development, we first injected pristane into *CD4^cre^Aim2^fl/fl^* and *Aim2^fl/fl^* mice (Figure 4A). In contrast with control mice, *CD4^cre^Aim2^fl/fl^* mice displayed reduced proteinuria (Figure 4B) and serum anti‐dsDNA antibody and ANA levels (Figure 4C) after pristane administration. Moreover, histological results showed less cell infiltration and C3 and IgG deposition in glomeruli in *CD4^cre^Aim2^fl/fl^* mice (Figure 4D). All these data suggested Aim2 deficiency in CD4^+^ T cells ameliorated lupus features in pristane‐induced lupus model. Given the aforementioned results that T_FH_‐AIM2 may have a function in SLE patients and deficiency of CD4^+^ T‐cell‐intrinsic Aim2 downregulates T_FH_ cell differentiation, we suspected that Aim2 deficiency could impair T_FH_ cell differentiation, which alleviated clinical lupus symptoms after pristane administration. Of note, we observed fewer T_FH_ cells in dLNs in *CD4^cre^Aim2^fl/fl^* mice than in *Aim2^fl/fl^* mice (Figure 4E). Likewise, a lower frequency of GC B cells was also identified in *CD4^cre^Aim2^fl/fl^* mice (Figure 4F). These data collectively showed that AIM2/Aim2 contributed to the SLE pathogenesis most likely by regulation of T_FH_ cell differentiation.

The cGVHD model is also a widely accepted lupus model (Figure S4A).^29^ To confirm our hypothesis of Aim2‐T_FH_ function in SLE, we therefore induced a cGVHD lupus model by transferring CD8^+^ T‐cell‐depleted lymphocytes from *Aim2^‐/‐^* or WT mice to B6D2F1 recipient mice. Similarly, mice receiving Aim2‐deficient cells showed milder clinical lupus symptoms, including less proteinuria (Figure S4B) and serum anti‐dsDNA antibody and ANA levels (Figure S4C). H&E‐stained kidney sections revealed less cell infiltration in glomeruli in mice receiving Aim2‐deficient cells (Figure S4D). In addition, poorer differentiation of T_FH_ cells and GC B cells was found in exogenous AIM2‐deficient recipient mice (Figure S4E and F). All these murine findings confirmed that loss of CD4^+^ T‐cell‐intrinsic Aim2 ameliorated lupus symptoms by regulating the T_FH_ response.

### 3.6 AIM2 regulates T_FH_ cell differentiation and interacts with c‐MAF

It has been proposed that AIM2 maybe resides in the nucleus of CD4^+^ T cells in the aforementioned human confocal assay (Figure 1F), which facilitates the potential interaction of AIM2 with other transcriptional factors. To better address the localization of AIM2 in CD4^+^ T cells, we performed cell fractionation followed by immunoblotting to assess the distribution of the AIM2 protein between the nuclear and cytoplasmic fraction in Aim2 knockout mice. Using *Aim2^‐/‐^* and WT mice, we observed that murine Aim2 accumulation mostly appeared in the nucleus but not the cytoplasm in splenic CD4^+^ T cells (Figure S3A). These observations collectively indicated that AIM2/Aim2 may reside in nucleus and have a function relating to transcriptional programs in T_FH_ cells, which most likely mediated T_FH_ cell development by cooperative transcriptional regulation.

**FIGURE 5:**
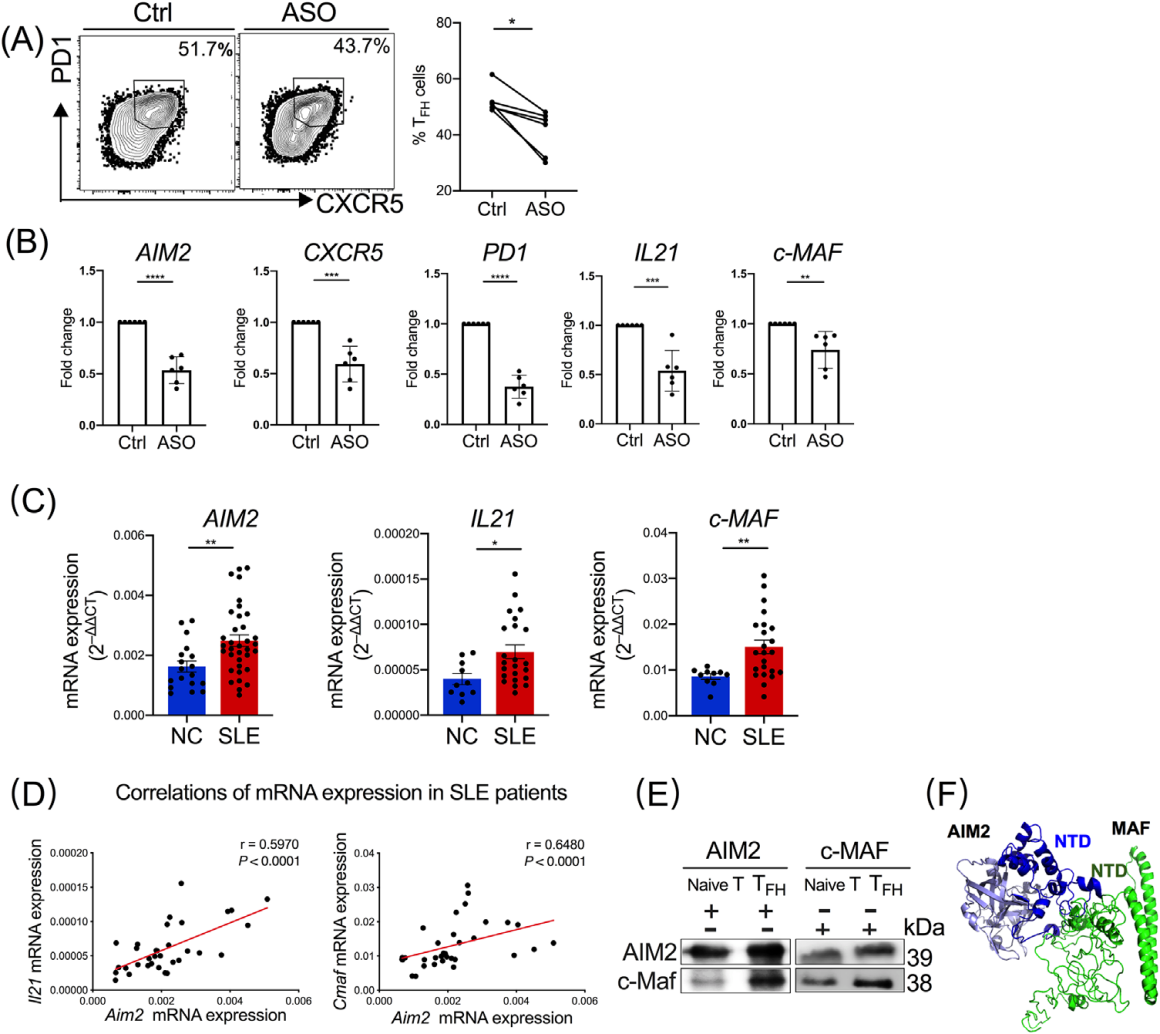
Absent in melanoma 2 (AIM2) regulates T follicular helper (T_FH_) cell differentiation and C‐Maf expression. (A and B) Antisense oligonucleotides of AIM2 and the control were transfected into naïve CD4^+^ T cells during T_FH_ cell differentiation for 5 days (*n* = 3). (A) Representative flow cytometric profiles and data plots of T_FH_ cells and (B) mRNA levels of *AIM2*, *CXCR5*, *PD1*, *IL21* and *c‐MAFmaf* detected by real‐time PCR are shown. (C) Real‐time PCR was performed to assess *AIM2*, *IL21* and *c‐MAF* mRNA levels in peripheral CD4^+^ T cells from systemic lupus erythematosus (SLE) patients and normal controls (*n* = 10 or 23, detailed information of the donors is shown in Table S5). (D) Correlations of mRNA expression of *Il21* and *Aim2* and of *Cmaf* and *Aim2* in (C). (E) Co‐immunoprecipitation of AIM2 with c‐Maf in naïve T and T_FH_ cells (*n* = 2). (F) The binding domains of c‐Maf‐AIM2. Bars show the mean ± SEM. *p < .05, **p < .01, ***p < .001

Accordingly, we next explored the potential mechanism of AIM2 in regulating T_FH_ cell response. To determine whether AIM2 regulates the differentiation of T_FH_ cells, we knocked down AIM2 by transferring ASOs during T_FH_ cell differentiation. Correspondingly, downregulated T_FH_ cell polarisation was observed in human AIM2‐knockdown cells (Figure 5A). Decreased transcriptomic expression of *AIM2* and T_FH_ ‐related genes, including *IL21* and *CXCR5*, in cells transfected with ASO was also observed, indicating that AIM2 may exert regulatory effects on T_FH_ cell differentiation (Figure 5B). Notably, the higher *c‐MAF* mRNA level in the control group suggested that c‐MAF production is enhanced in the regulation of AIM2 during T_FH_ cell differentiation. In addition, in contrast to NCs, we found higher mRNA expression of *AIM2*, *IL21* and *c‐MAF* in CD4^+^ T cells from peripheral blood in SLE patients (Figure 5C). Positive correlations were further observed in the mRNA expression between *AIM2* and *Il21* and between *AIM2* and *c‐MAF* in SLE (Figure 5D). The role of IL‐21‐c‐MAF in regulating T_FH_ cell development has been well established.^7^ As the activator of IL‐21 promoter and enhancer, c‐MAF is crucial for producing and sustaining IL‐21, which largely promotes T_FH_ cell differentiation and GC formation, consequently resulting in enriched c‐MAF levels and T_FH_ cell response. These findings suggested that the regulation of AIM2 in T_FH_ cell differentiation may be responsible for SLE progression, which was mediated by the IL‐21‐c‐MAF pathway in T_FH_ cells. Hence, we speculated whether AIM2 can bind to c‐MAF, which then contributed to orchestrated T_FH_ cell signalling pathways in SLE. To explore the interaction between AIM2 and c‐MAF, we performed Co‐IP assays and confirmed that AIM2 did bind directly with c‐MAF in human T_FH_ cells (Figure 5E). Candidate interaction domains for c‐MAF with AIM2 were also predicted by homology‐modelling supported structural analysis (Figure 5F). Taken together, these data suggested AIM2 regulated T_FH_ cell differentiation in SLE by interaction with c‐MAF and mediation in IL‐21‐c‐MAF signalling pathway.

### 3.7 IL‐21 promotes AIM2 expression by recruiting TET2 to the *AIM2* promoter

**FIGURE 6:**
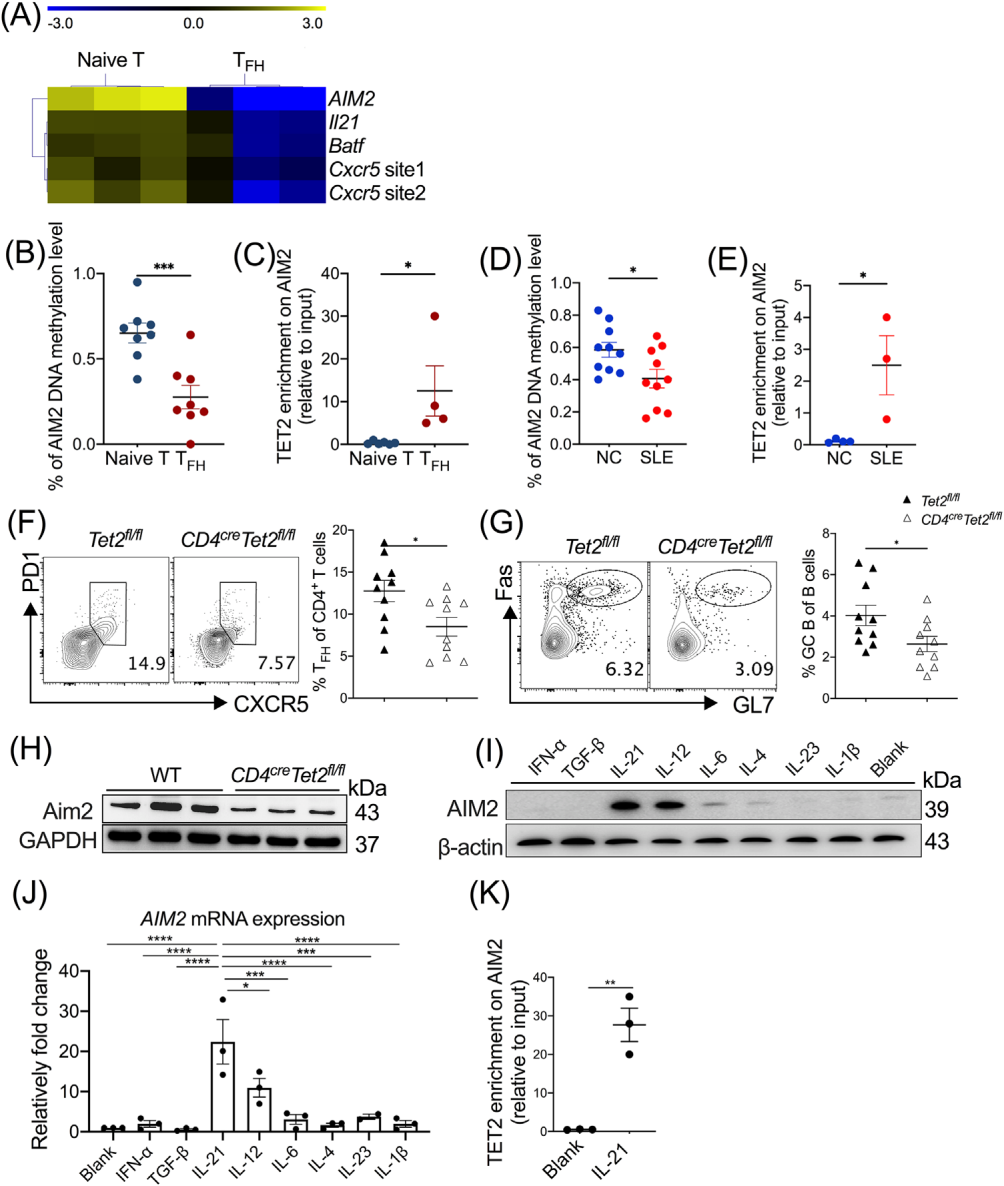
IL‐21 positively regulates absent in melanoma 2 (AIM2) expression by promoting ten‐eleven translocation 2 (TET2) enrichment in the *Aim2* promoter region of T follicular helper (T_FH_) cells. (A) DNA methylation map of naïve CD4^+^ T cells and T_FH_ cells (*n* = 3). Bisulphite sequencing PCR was performed to assess DNA methylation levels in the Aim2 promoter of (B) naïve CD4^+^ T cells and T_FH_ cells (*n* = 8) and (D) peripheral CD4^+^ T cells from systemic lupus erythematosus (SLE) patients and normal controls (*n* = 10). Chromatin immunoprecipitation was performed to assess TET2 enrichment in the *Aim2* promoter of (C) naïve CD4^+^ T cells and T_FH_ cells (*n* = 4 or 6) and (E) peripheral CD4^+^ T cells from SLE patients and normal controls (*n* = 3 or 4). Representative flow cytometric profiles and data plots of (F) T_FH_ cells and (G) germinal centre (GC) B cells in draining lymph nodes (dLNs) from *Tet2^fl/fl^* and *CD4^cre^Tet2^fl/f^* ^l^ mice immunised with keyhole limpet haemocyanin (KLH) (*n* = 9 or 10). (H) AIM2 accumulation in CD4^+^ T cells in wild‐type (WT) and *CD4^cre^Tet2^fl/fl^* mice (*n* = 3). AIM2 accumulation in response to treatments with T_FH_‐related cytokines assessed by (I) Western blot and (J) real‐time PCR (*n* = 3). (K) Chromatin immunoprecipitation was performed in human CD4^+^ T cells with or without IL‐21 treatment after TET2 pull‐down (*n* = 3). Bars show the mean ± SEM. *p < .05, **p < .01, ***p < .001, ****p < .0001

The epigenetic machinery is well recognised in the modulation of T_FH_ cell differentiation and pathogenesis of SLE.^17^, ^30^, ^31^ To test the methylation levels of AIM2 in T_FH_ cell differentiation, we first performed DNA methylation sequencing. As showed in the DNA methylation map (Figure 6A), T_FH_ cells, in contrast to naïve T cells, showed decreased DNA methylation levels in the *AIM2* promoter region, which was also validated by bisulphite genomic sequencing (Figure 6B). As a hydroxymethyltransferase, TET2 leads to DNA demethylation by catalysing 5mC to 5hmC of targeted genes, which contributes to the regulation of T‐cell lineage differentiation and SLE development.^30^ In our study, we suspected TET2 might mediate aberrant methylation levels of AIM2 and enhance SLE development. To test this, we explored the interaction of TET2 with AIM2 by performing ChIP assay. As showed in Figure 6C, increased enrichment of TET2 in the *AIM*2 promoter was observed in T_FH_ cells but not naïve T cells, which suggested that epigenetic modulation of AIM2 in T_FH_ cells is mediated by TET2. Significantly, SLE patients showed decreased methylation levels of the *AIM*2 promoter (Figure 6D) and upregulated enrichment of TET2 on the *AIM*2 promoter (Figure 6E). In an in vivo system, *CD4^cre^Tet2^fl/fl^* mice likewise showed reduced percentages of T_FH_ and GC B cells (Figure 6F and G) in dLNs and reduced accumulation of Aim2 protein in CD4^+^ T cells upon KLH immunisation (Figure 6H). These human and murine data showed that TET2 can mediate demethylation of *AIM2*, which may be responsible for increased T_FH_ cell differentiation and enhancing SLE pathogenesis.

To investigate the probable upstream modulation of TET2‐AIM2 pathway in the T_FH_ cells, we then screened all known cytokines required for human T_FH_ cell differentiation. IL‐21 dramatically increased AIM2 expression during in vitro differentiation at the protein and mRNA levels (Figure 6I and J), which may be the upstream regulator of the AIM2‐T_FH_ pathway. Further ChIP‐PCR analysis confirmed higher transcriptomic *AIM2* levels of IL‐21‐stimulated CD4^+^ T cells after TET2 pull‐down (Figure 6K), indicating that TET2 might be a DNA demethylation‐based on‐ switch for AIM2 transcription. Collectively, these results suggested that IL‐21 increased AIM2 expression by inducing TET2 enrichment in the *AIM2* promoter, leading to a reduced DNA methylation level of the *AIM2* locus and enriched T_FH_ cells, which can promote the development of SLE.

## 4 DISCUSSION

AIM2 is an important inflammatory DNA sensor in innate immunity. However, the function of AIM2 has seldom been revealed in T cells. A recent study reported that the inflammasome‐independent role of Aim2‐attenuated EAE symptoms via metabolic regulation of switching glycolysis to oxidative phosphorylation in Treg cells.^32^ Here, we have identified a previously unrecognised function of AIM2 in SLE pathogenesis. We observed T_FH_ cells expressing higher AIM2 levels than other CD4^+^ T‐cell subtypes in both the human secondary lymphoid organ and in vitro differentiation assays. Additionally, we found higher expression of AIM2‐T_FH_ in SLE patients and further confirmed that CD4^+^ T cell‐Aim2 accelerates lupus symptoms mediated by regulation of T_FH_ cell differentiation in disease models. Moreover, decreased methylation levels of *AIM2* in CD4^+^ T cells were found in SLE patients compared with normal healthy people. IL‐21 promoted AIM2 expression by enhancing TET2 recruitment to the *AIM2* promoter. AIM2 might regulate c‐MAF production, thereby further regulating IL‐21 expression and T_FH_ cell differentiation.

AIM2‐like receptors, including IFN‐inducible protein 16 and AIM2, are gaining increasing attention in SLE due to the significant pathogenic role of IFN in lupus and the positive association between disease activity in SLE and inflammasome‐induced production of IL‐1β.^33^, ^34^ In lupus macrophages, AIM2 has been found to be directly activated by cytoplasmic DNA, consequently activating inflammasome responses and cell death.^35^, ^36^ SLE is characterised by an elevated apoptosis rate and defective clearance of ds‐DNAs by macrophages.^37^ Here, our study directly linked AIM2 with a prevalent autoimmune disorder.

In our study, AIM2 increased along with T_FH_ cell differentiation. T_FH_ cells promote B‐cell differentiation and maturation with the help of crosstalk of costimulatory molecules (ICOS, CD40L, TCR) and regulatory cytokines such as IL‐21.^38^ In addition to BCL6, c‐MAF is also well acknowledged as a transcription factor that contributes to T_FH_ cells.^39^ Previous work revealed that c‐MAF commits itself to T_FH_ cell differentiation by directly enhancing the production of IL‐21 and CXCR5 and the promotion of CXCR4, PD1 and ICOS by cooperating with BCL6.^40^ AIM2 promotes the production of c‐MAF and T_FH_ cell differentiation, resulting in increased levels of IL‐21. TET2 proteins function in DNA demethylation by catalysing the conversion from 5mC to 5hmC. Studies have indicated that TET2 recruits IL‐6 and IFN‐γ, lineage‐specific cytokine genes, to control the methylation levels and cell differentiation of Th1 and T_H_17 cells.^41^ Early findings also suggested that increased *Bcl6* transcription was related to decreased levels of 5hmC in T_FH_ cells.^21^ Our previous studies revealed that TET2 enrichment on the *BCL6* promoter was attributed to IL‐21, which potentially explains why lupus T cells have higher BCL6 expression.^5^ In the present study, TET2 was enriched in the *Aim2* promoter after IL‐21 stimulation, indicating that a DNA demethylation‐based regulatory mechanism may underlie our observation of increased AIM2 levels upon IL‐21 treatment.

The crucial function of AIM2 in tumour and innate immune response is well established.^42^, ^43^ However, our study suggested an important role of AIM2 in adaptive immune cells (T_FH_ cells) in a classical autoimmune disease model. The highly expressed AIM2 level and increased AIM2‐T_FH_ cells in SLE but not in other diseases like psoriasis or healthy people, suggested a potential of AIM2 as a biological marker in the diagnosis of SLE. Furthermore, patients with DLE (a subtype of cutaneous lupus erythematosus) whose main manifestation was skin lesions, displayed much more increased AIM2 levels than patients with SLE, suggesting the clinical implication of AIM2, particularly in skin lesion features of lupus patients. Further study may focus on whether AIM2 can promote the inflammatory cell infiltration in skin and our study paved the way for the potential of AIM2‐targeted treatment in skin lesions of lupus patients.

Although significant advances in B‐cell‐targeted therapy for the treatment of SLE have been achieved, extensive studies have suggested a key role of effector T‐cell subsets in lupus pathogenesis.^44^ Here, our findings demonstrated that high AIM2 expression upregulates the T_FH_ cell response and that the IL‐21‐AIM2‐TET2‐c‐MAF pathway contributes to autoimmune progression during lupus development, which may facilitate new clinical therapeutics targeting AIM2 in T cells for the treatment of SLE.

## Supporting information

Supporting Information
