# Supplementary material for: The IL‐21‐TET2‐AIM2‐c‐MAF pathway drives the T follicular helper cell response in lupus‐like disease
Source: Clin Transl Med. 2022 Mar 28;12(3):e781. doi: 10.1002/ctm2.781 (PMC8958352; doi:10.1002/ctm2.781)
Supplement: Supplementary file 1 — Supporting Information [file CTM2-12-e781-s001.docx]

**Supplementary Figures**

**
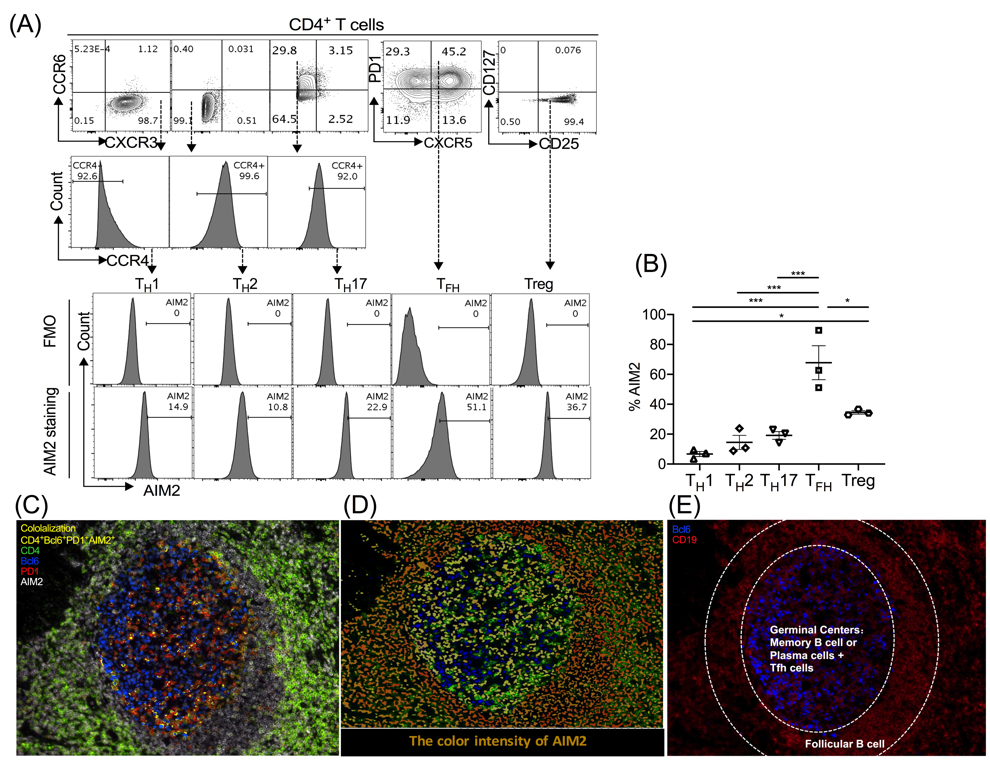
**

**FIGURE S1:** **AIM2 is highly expressed in human T_FH_ cells.**

(A-B) In vitro-differentiation of various CD4^+^ T cell subsets, was performed using human peripheral cells. (A) Gating strategy and (B) representative flow cytometric plots of AIM2 percentages among various CD4^+^ T cell subsets were shown (n=3). (C-E) Multicolor IHC was performed on human tonsil tissues. (C) The co-localization of AIM2 and T_FH_-like cells, (D) the expressive intensity of AIM2, and (E) the distribution of B cells in the germinal center from human tonsil tissues were shown. Bars show the mean ± SEM. * *P* < 0.05. ** *P* < 0.01, *** *P* < 0.001, **** *P* < 0.0001.


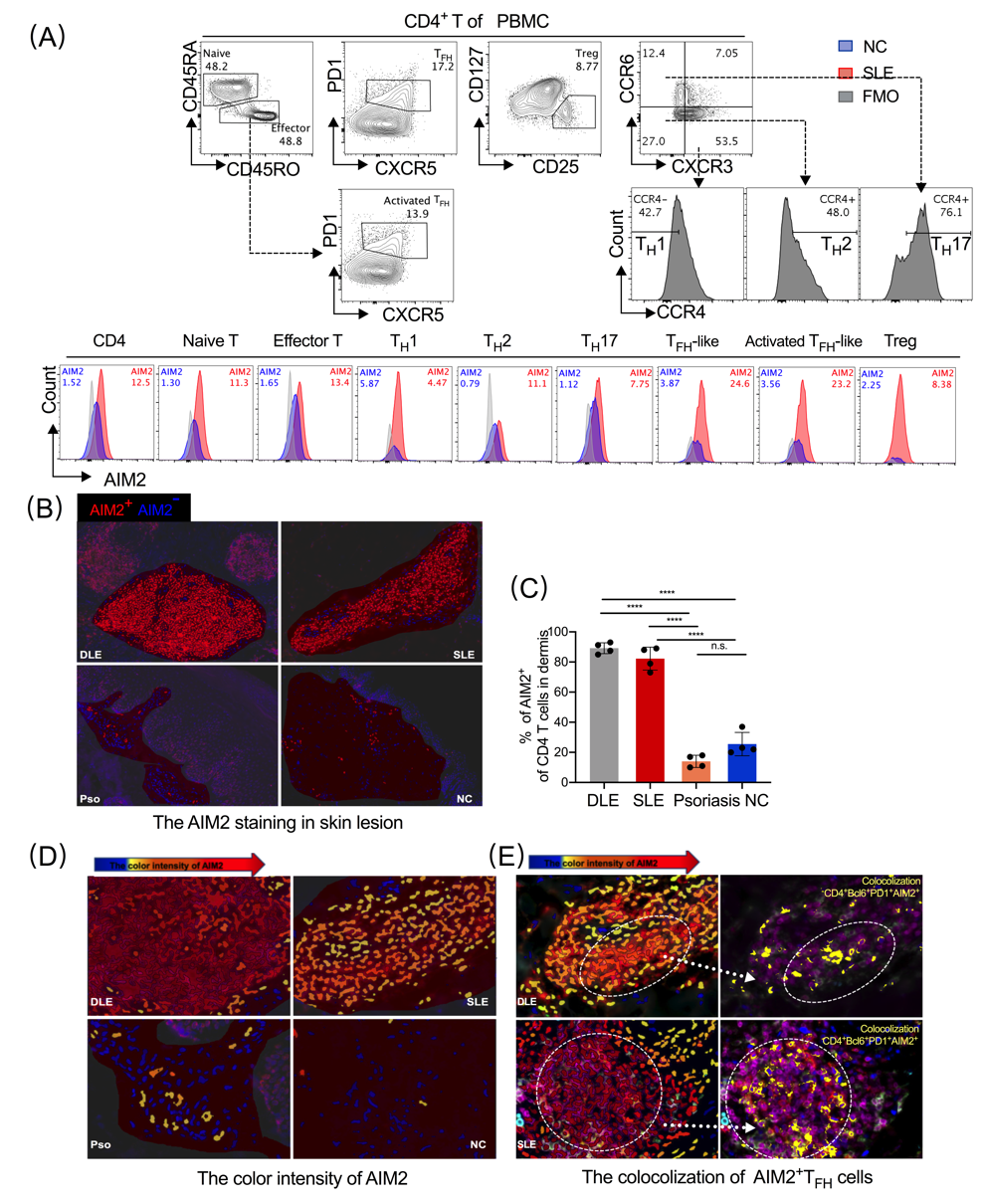


**FIGURE S2:** **AIM2 is highly expressed in T_FH_-like cells from peripheral blood and skin lesions in SLE patients.**

(A) Gating strategy and representative flow cytometric plots of AIM2 expression in various CD4^+^ T cell subsets in peripheral blood from SLE patients and normal controls were shown **(**n=19**).** (B-E) Multicolor IHC of skin lesions from patients with SLE, DLE, psoriasis, and normal control was performed (n=4). (B) The representative images and (C) quantified data plots of AIM2 expression in infiltrating cells from (B) were shown. (D) The expressive intensity of AIM2 of skin lesions from patients with SLE, DLE, psoriasis, and normal control. (E) The expressive intensity of AIM2 (left) and co-localization of AIM2 and T_FH_-like cells in lupus patients (right) were shown. Bars show the mean ± SEM. * *P* < 0.05. ** *P* < 0.01, *** *P* < 0.001, **** *P* < 0.0001.

**
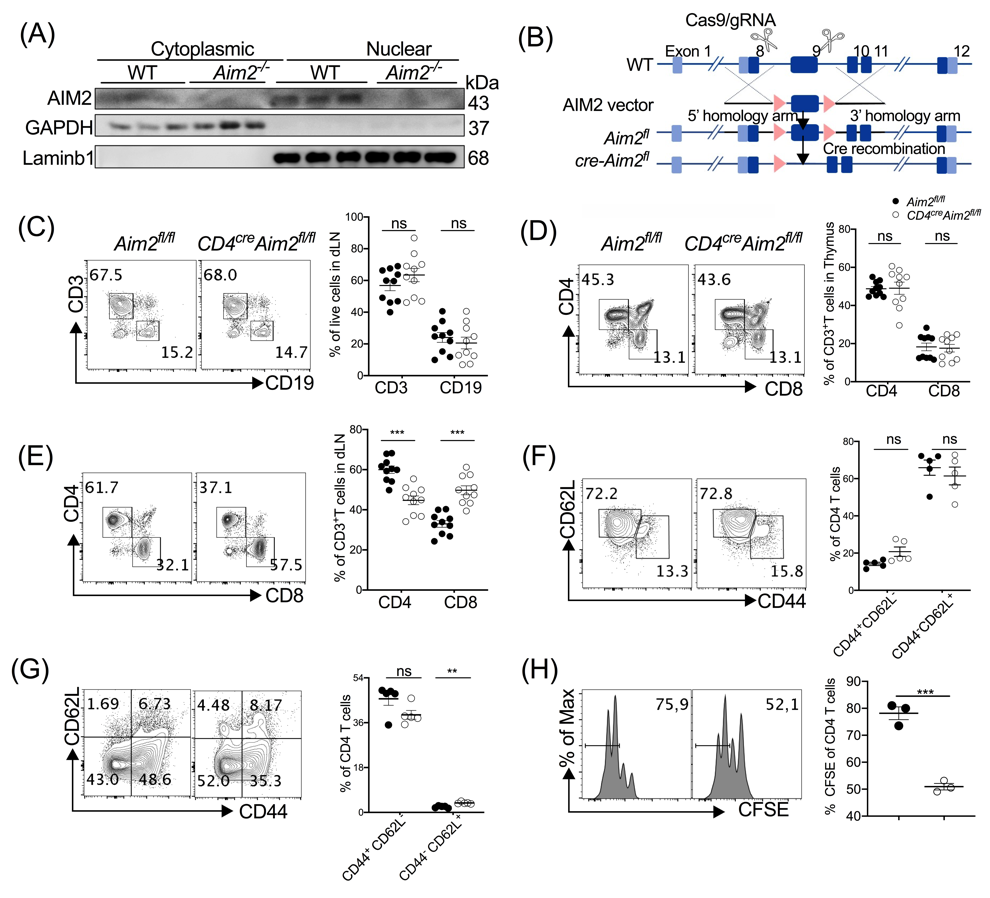
**

**FIGURE S3:** **Reduced proliferation in AIM2 deficient CD4^+^ T cells**

(A) Nuclear and cytoplasmic expression of AIM2 in splenic CD4^+^ T cells of *Aim2^-/-^* and WT mice was shown (n = 3). (B) Schematic of generating *CD4^cre^Aim2^fl/fl^* mice. (C-F) Cells from *Aim2^fl/fl^* and *CD4^cre^Aim2^fl/fl^* mice at steady state were analyzed by flow cytometry. The representative flow cytometric plots and data plots of (C) T cells (CD3^+^) and B cells (CD19^+^) from draining lymph nodes (n=10), CD4^+^ T and CD8^+^ T cells from (D) thymus and (E) draining lymph nodes (n=10), and (F) effector T (CD44^+^CD62L^-^) and naïve CD4^+^ T (CD44^-^CD62L^+^) cells in the draining lymph nodes (n = 5) were shown. (G-H) Splenic CD4^+^ T cells from *Aim2^fl/fl^* mice and *CD4^cre^Aim2^fl/fl^* mice were treated with anti-CD3e and anti-CD28 in culture for 3 days. The representative flow cytometric plots and data plots of (G) naïve CD4^+^ T and effector CD4^+^ T cells (n = 5), and (H) proliferated CFSE-CD4^+^ T cells (n = 3) from *CD4^cre^Aim2^fl/fl^* mice and *Aim2^fl/fl^* mice were shown. Bars show the mean ± SEM. * *P* < 0.05. ** *P* < 0.01, *** *P* < 0.001, **** *P* < 0.0001.


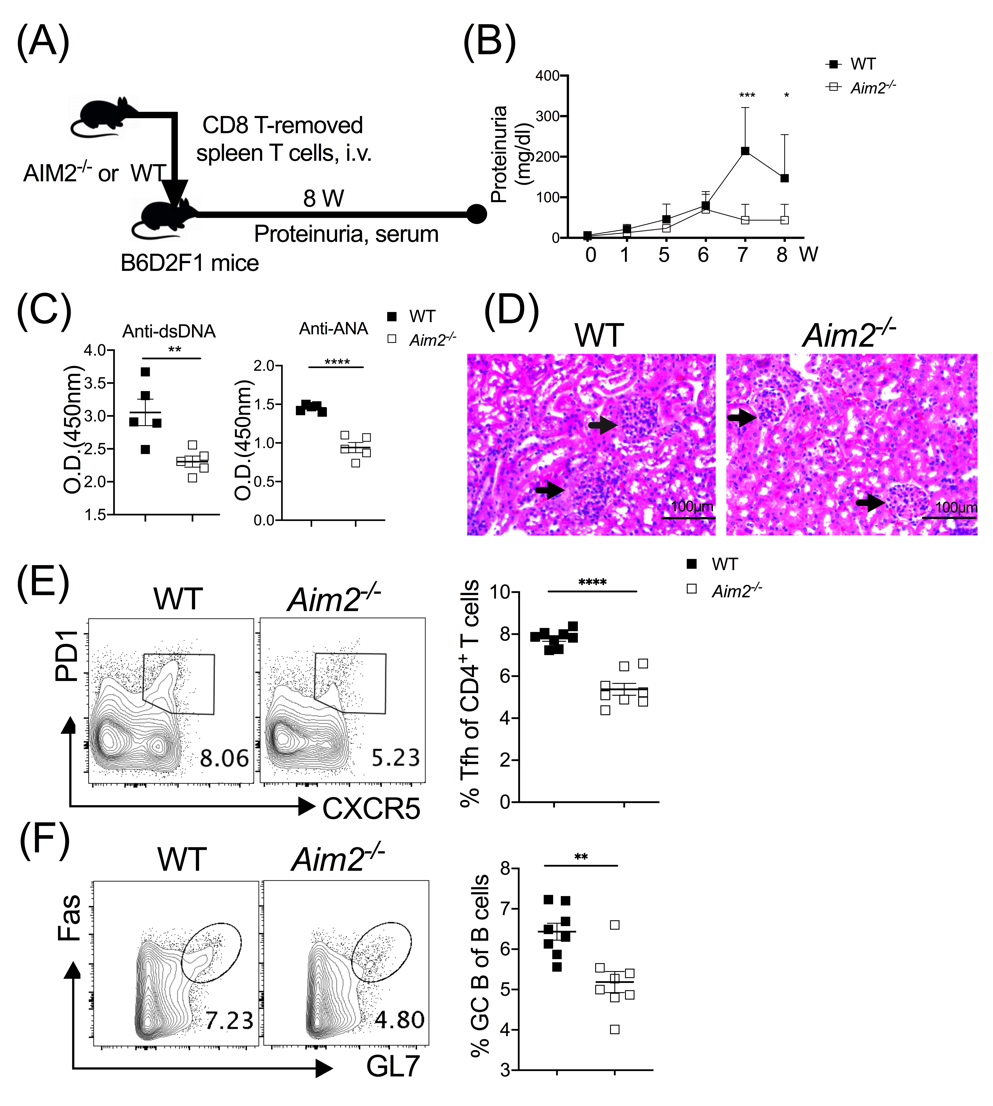


**FIGURE S4: Aim2 deficiency in CD4^+^ T cells ameliorates lupus development in mice.**

The chronic graft versus host disease (cGVHD) lupus model was induced by injecting splenic CD8^+^ T-depleted cells from WT and *Aim2^-/-^* mice to B6D2F1 mice. (A) Schematics of cGVHD-lupus model. (B) Proteinuria of mice, and (C) sera levels of anti-dsDNA and anti-ANA antibodies at the end of the model were shown. (D) Representative images of H&E-stained kidney glomeruli were shown. The representative flow cytometric plots and data plots of (E) T_FH_ (CXCR5^+^PD1^+^CD4^+^) and (F) GC B (GL7^+^Fas^+^B220^+^) cells in draining lymph nodes were shown. Horizontal bars represent the mean ± SEM. Bars show the mean ± SEM. * *P* < 0.05. ** *P* < 0.01, *** *P* < 0.001, **** *P* < 0.0001.

**TABLE S1:** Demographic data for each participant in Multicolor IHC staining

*DLE, discoid lupus erythematosus; SLE, systemic lupus erythematosus; Pso, psoriasis; NC, normal control; N.A., not applicable.

| Sample ID | Sex | Age(year) | SLEDAI score | |
| --- | --- | --- | --- | --- |
| DLE1 | F | 43 | N.A. |  |
| DLE2 | M | 29 | N.A. |  |
| DLE3 | F | 21 | N.A. |  |
| DLE4 | M | 67 | N.A. |  |
| SLE1 | F | 33 | 12 |  |
| SLE2 | F | 35 | 18 |  |
| SLE3 | F | 41 | 8 |  |
| SLE4 | M | 34 | 16 |  |
| Pso1 | F | 22 | N.A. |  |
| Pso2 | M | 46 | N.A. |  |
| Pso3 | M | 41 | N.A. |  |
| NC1 | F | 38 | N.A. |  |
| NC2 | M | 30 | N.A. |  |
| NC3 | F | 27 | N.A. |  |
| NC4 | F | 47 | N.A. |  |

**TABLE S2:** Demographic data for each participant in tonsil assessment by flow cytometry and multicolor IHC

| Tonsil ID | | Sex | Age (year) | Diagnosis |
| --- | --- | --- | --- | --- |
| 1 | M | | 5 | Chronic tonsillitis |
| 2 | F | | 34 | Chronic tonsillitis |
| 3 | M | | 42 | Chronic tonsillitis |
| 4 | F | | 7 | Tonsil hypertrophy |
| 5 | M | | 5 | Tonsil hypertrophy |
| 6 | M | | 22 | Chronic tonsillitis |
| 7 | M | | 27 | Chronic tonsillitis |
| 8 | F | | 42 | Chronic tonsillitis |
| 9 | M | | 8 | Chronic tonsillitis |
| 10 | F | | 19 | Chronic tonsillitis |
| 11 | M | | 13 | Chronic tonsillitis |
| 12 | F | | 8 | Chronic tonsillitis |
| 13 | F | | 10 | Chronic tonsillitis |

**TABLE S3:** Demographic data for each participant in assessment of peripheral blood by flow cytometry, confocal and western blot

* SLE, systemic lupus erythematosus; NC, normal control.

Participants in flow cytometry

| Sample | Sex | Age | SLEDAI score |
| --- | --- | --- | --- |
| SLE 5 | F | 42 | 2 |
| SLE 6 | F | 25 | 6 |
| SLE 7 | F | 38 | 8 |
| SLE 8 | F | 38 | 6 |
| SLE 9 | F | 27 | 2 |
| SLE 10 | F | 49 | 0 |
| SLE 11 | F | 46 | 2 |
| SLE 12 | F | 17 | 8 |
| SLE 13 | F | 26 | 0 |
| SLE 14 | F | 41 | 6 |
| SLE 15 | M | 47 | 2 |
| SLE 16 | F | 31 | 2 |
| SLE 17 | M | 56 | 6 |
| SLE 18 | F | 53 | 6 |
| SLE 19 | F | 46 | 0 |
| SLE 20 | F | 25 | 8 |
| SLE 21 | F | 15 | 6 |
| SLE 22 | F | 28 | 12 |
| SLE 23 | F | 46 | 2 |
| NC 5 | F | 57 | N.A. |
| NC 6 | F | 48 | N.A. |
| NC 7 | F | 48 | N.A. |
| NC 8 | F | 45 | N.A. |
| NC 9 | F | 40 | N.A. |
| NC 10 | F | 47 | N.A. |
| NC 11 | F | 50 | N.A. |
| NC 12 | F | 39 | N.A. |
| NC 13 | F | 41 | N.A. |
| NC 14 | F | 58 | N.A. |
| NC 15 | F | 54 | N.A. |
| NC 16 | F | 42 | N.A. |
| NC 17 | F | 54 | N.A. |
| NC 18 | F | 62 | N.A. |
| NC 19 | F | 56 | N.A. |
| NC 20 | F | 58 | N.A. |
| NC 21 | F | 48 | N.A. |
| NC 22 | F | 43 | N.A. |
| NC 23 | F | 49 | N.A. |
| NC 24 | F | 38 | N.A. |
| NC 25 | F | 58 | N.A. |
| NC 26 | F | 40 | N.A. |
| NC 27 | F | 37 | N.A. |
| NC 28 | F | 59 | N.A. |
| NC 29 | F | 55 | N.A. |
| NC 30 | F | 49 | N.A. |
| NC 31 | F | 59 | N.A. |
| NC 32 | F | 31 | N.A. |
| NC 33 | F | 25 | N.A. |

Participants in confocal

| Sample | Sex | Age | SLEDAI score SCORE |
| --- | --- | --- | --- |
| SLE 24 | F | 27 | 4 |
| SLE 25 | F | 33 | 0 |
| SLE 26 | F | 24 | 2 |
| NC 34 | F | 36 | N.A. |
| NC 35 | F | 31 | N.A. |
| NC 36 | F | 19 | N.A. |

Participants in western blot

| Sample | Sex | Age | SLEDAI score |
| --- | --- | --- | --- |
| SLE 27 | F | 37 | 0 |
| SLE 28 | F | 25 | 1 |
| SLE 29 | F | 14 | 0 |
| SLE 30 | F | 22 | 4 |
| NC 37 | F | 19 | N.A. |
| NC 38 | F | 23 | N.A. |
| NC 39 | F | 31 | N.A. |
| NC 40 | F | 26 | N.A. |

**TABLE S4:** Demographic data for each participant in assessment of skin lesions by RT-PCR

* NC, normal control; N.A., not applicable; DLE, discoid lupus erythematosus; SCLE, subacute cutaneous lupus erythematosus; ACLE, acute cutaneous lupus erythematosus.

| Sample ID | Sex | Age | Diagnosis |
| --- | --- | --- | --- |
| Lupus 1 | M | 38 | DLE |
| Lupus 2 | M | 29 | SCLE |
| Lupus 3 | F | 46 | SCLE |
| Lupus 4 | F | 33 | DLE |
| Lupus 5 | F | 12 | ACLE |
| Lupus 6 | M | 86 | DLE |
| Lupus 7 | M | 47 | DLE |
| Lupus 8 | F | 36 | SCLE |
| Lupus 9 | F | 26 | ACLE |
| Lupus 10 | F | 61 | DLE |
| Lupus 11 | F | 34 | ACLE |
| Lupus 12 | F | 66 | ACLE |
| Lupus 13 | F | 24 | DLE |
| Lupus 14 | F | 21 | DLE |
| Lupus 15 | F | 30 | ACLE |
| Lupus 16 | M | 66 | DLE |
| Lupus 17 | M | 72 | SCLE |
| Lupus 18 | F | 33 | ACLE |
| Lupus 19 | F | 28 | ACLE |
| Lupus 20 | F | 32 | SCLE |
| NC 41 | F | 53 | N.A. |
| NC 42 | M | 66 | N.A. |
| NC 43 | M | 32 | N.A. |
| NC 44 | M | 52 | N.A. |
| NC 45 | F | 49 | N.A. |
| NC 46 | F | 52 | N.A. |
| NC 47 | F | 24 | N.A. |
| NC 48 | F | 43 | N.A. |
| NC 49 | F | 28 | N.A. |
| NC 50 | F | 43 | N.A. |
| NC 51 | F | 48 | N.A. |
| NC 52 | M | 74 | N.A. |
| NC 53 | M | 23 | N.A. |
| NC 54 | F | 52 | N.A. |
| NC 55 | F | 29 | N.A. |
| NC 56 | F | 63 | N.A. |
| NC 57 | F | 40 | N.A. |
| NC 58 | M | 13 | N.A. |
| NC 59 | F | 18 | N.A. |
| NC 60 | F | 17 | N.A. |

**TABLE S5:** Demographic data for each participant in assessment of peripheral blood by RT-PCR.

* SLE, systemic lupus erythematosus, NC, normal control; N.A., not applicable.

| Sample | Sex | Age | SLEDAI score |
| --- | --- | --- | --- |
| SLE 31 | M | 48 | 6 |
| SLE 32 | F | 47 | 2 |
| SLE 33 | F | 44 | 8 |
| SLE 34 | F | 46 | 4 |
| SLE 35 | F | 40 | 6 |
| SLE 36 | F | 40 | 4 |
| SLE 37 | F | 41 | 10 |
| SLE 38 | F | 38 | 7 |
| SLE 39 | F | 47 | 3 |
| SLE 40 | M | 37 | 8 |
| SLE 41 | F | 26 | 22 |
| SLE 42 | F | 37 | 14 |
| SLE 43 | F | 37 | 13 |
| SLE 44 | M | 63 | 11 |
| SLE 45 | F | 50 | 12 |
| SLE 46 | F | 65 | 10 |
| SLE 47 | F | 35 | 24 |
| SLE 48 | F | 31 | 2 |
| SLE 49 | F | 38 | 12 |
| SLE 50 | F | 20 | 10 |
| SLE 51 | F | 24 | 8 |
| SLE 52 | F | 30 | 2 |
| NC 61 | F | 24 | N.A. |
| NC 62 | F | 24 | N.A. |
| NC 63 | F | 22 | N.A. |
| NC 64 | F | 23 | N.A. |
| NC 65 | M | 30 | N.A. |
| NC 66 | F | 42 | N.A. |
| NC 67 | F | 24 | N.A. |
| NC 68 | F | 22 | N.A. |
| NC 69 | F | 25 | N.A. |

**TABLE S6:** Demographic data for each participant in assessment of peripheral blood by mRNA-seq analysis and RT-PCR.

NC, normal control; N.A., not applicable.

| Sample | Sex | Age | SLEDAI score |
| --- | --- | --- | --- |
| NC 70 | F | 28 | N.A. |
| NC 71 | F | 41 | N.A. |
| NC 72 | F | 30 | N.A. |
| NC 73 | F | 32 | N.A. |
| NC 74 | F | 26 | N.A. |
| NC 75 | M | 45 | N.A. |
| NC 76 | F | 37 | N.A. |
| NC 77 | F | 29 | N.A. |
| NC 78 | F | 23 | N.A. |
| NC 79 | F | 27 | N.A. |

**TABLE S7:** Antibodies used for flow cytometry, confocal, multicolor IHC and western blot.

| Antigen | Dilution | Cat. number | Source |
| --- | --- | --- | --- |
| CD4 | 1：200 | ab214411 | Abcam |
| Bcl-6 | 1：100 | 14895 | Cell signaling Technology |
| Stat3 | 1：100 | 12640 | Cell signaling Technology |
| c-Maf | 1：100 | ab230928 | Abcam |
| AIM2 | 1：100 | ab204995 | Abcam |
| DyLight 594 IgG | 1：100 | A23420 | Abbkine |
| DyLight 488 IgG | 1：100 | A23210 | Abbkine |
| C3 | 1：100 | ab11862 | Abcam |
| IgG | 1：100 | ab150117 | Abcam |
| CD4 | 1：100 | RMA-0620 | MXB Biotechnologies |
| PD1 | 1：100 | ab137132 | Abcam |
| Bcl6 | 1：100 | ab33901 | Abcam |
| CXCR5 | 1：100 | ab46218 | Abcam |
| CD19 | 1：100 | ab195896 | Abcam |
| AIM2 | 1：100 | ab93015 | Abcam |
| CD4 | 1：200 | 555349 | BD Biosciences |
| CXCR5 | 1：200 | 356926 | Biolegend |
| CD127 | 1：200 | 351322 | Biolegend |
| CD25 | 1：200 | 302636 | Biolegend |
| CD45RA | 1：200 | 304140 | Biolegend |
| CD45RO | 1：200 | 17-0457-42 | eBioscience |
| CXCR3 | 1：200 | 353716 | Biolegend |
| CCR6 | 1：200 | 353430 | Biolegend |
| CCR4 | 1：200 | 359418 | Biolegend |
| AIM2 | 1：200 | 652804 | Biolegend |
| CD8a | 1：200 | 300920 | Biolegend |
| CD3 | 1：200 | 200470 | Biolegend |
| CD62L | 1：200 | 304822 | Biolegend |
| CD4 | 1：200 | 552051 | BD Biosciences |
| CD16/CD32 | 1：250 | 101320 | BioLegend |
| CXCR5 | 1：200 | 560617 | BD Biosciences |
| CD279 | 1：200 | 262671 | BD Biosciences |
| AIM2 | 1：1000 | ab204995 | Abcam |
| AIM2 | 1：1000 | 63660 | Cell signaling Technology |
| β-actin | 1：5000 | sc-47778 | Santa Cruz |
| LaminB1 | 1：12000 | 13435S | Cell signaling Technology |
| GAPDH | 1：5000 | sc-32233 | Santa Cruz |

**TABLE S8:** Primer sequences and siRNA sequences

| **Quantitative PCR primers** | |
| --- | --- |
| Human-AIM2-Foward | ATGTGAAGCCGTCCAGA |
| Human-AIM2-Reverse | CATCATTTCTGATGGCTGCA |
| Human-CXCR5-Foward | GCACCTCCCATCCTAATCATC |
| Human-CXCR5-Reverse | CTAAGCTGATGGAGTGTGTTCT |
| Human-PD1-Foward | CCCTGGTGGTTGGTGTCGT |
| Human-PD1-Reverse | GCCTGGCTCCTATTGTCCCTC |
| Human-Cmaf-Foward | TGCACTTCGACGACCGCTTCTC |
| Human-Cmaf-Reverse | CGCTGCTCGAGCCGTTTTCTC |
| Human-Bcl6-Foward | CCCAAGGAAACAATCCCAGAAGAG |
| Human-Bcl6-Reverse | CTCATCTTCCGAGGAGGGTCTC |
| Human-β-actin-Foward | GAGCTACGAGCTGCCTGACG |
| Human-β-actin-Foward | GTAGTTTCGTGGATGCCACAG |
| **ChIP-qPCR primers** | |
| AIM2-Forward | CAAGAAGTCATCTGCGGTCA |
| AIM2-Reverse | CTGAGCTGGTTTGGGTTCTC |
| **BSP primers** | |
| First nested AIM2-Foward | TGGTTGAGTTGGTTTGGGTT |
| First nested AIM2-Reverse | TCACCAAACCCCTTAATCACA |
| Second nested AIM2-Foward | AGGTTTAGGTTTTTAGAGGTGTGT |
| Second nested AIM2-Reverse | TTCCGAAAATTACCCGCCCA |
| **ASO target sequence** | |
| AIM2 ASO | GATCAACACGCTTCAAACTC |
